# A novel method to reduce noise in electroretinography using skin electrodes: a study of noise level, inter-session variability, and reproducibility

**DOI:** 10.1007/s10792-016-0240-5

**Published:** 2016-06-09

**Authors:** Tsutomu Yamashita, Atsushi Miki, Akio Tabuchi, Hideaki Funada, Mineo Kondo

**Affiliations:** 1 Department of Sensory Science, Faculty of Health Science and Technology, Kawasaki University of Medical Welfare, 288 Matsushima, Kurashiki, 701-0193 Japan; 2 Department of Ophthalmology, Kawasaki Medical School, Kurashiki, Japan; 3 Tomey Corporation, Nagoya, Japan; 4 Department of Ophthalmology, Mie University Graduate School of Medicine, Tsu, Japan

**Keywords:** Electroretinogram (ERG), Skin electrode, Noise reduction, Noise level, Reproducibility, Variability

## Abstract

To determine the feasibility of recording reproducible electroretinograms (ERGs) with skin electrodes using a new ERG system. Seventeen healthy volunteers were studied. The dark-adapted, bright-flash ERGs were recorded with a new ERG recording system (LE-4000, Tomey, Nagoya, Japan) in which the stimulus alternated between the eyes every 15 s, and each eye was stimulated eight times. The active skin electrode was placed on the lower eyelids of both eyes. The voltage changes of the non-stimulated eye were subtracted from that of the stimulated eye to try to increase the signal-to-noise ratio for eight stimulus cycles. The noise levels were measured from 12 subjects with and without the subtraction steps. ERGs were also recorded on five different days from five subjects, and the coefficient of variation (CV) and the intra-class correlation coefficients (ICCs) were calculated. The noise level without the subtraction step was 18.4 ± 8.4 μV, and it was significantly reduced to 13.8 ± 4.0 μV with the subtraction step (*P* = 0.001). Reproducible ERGs were obtained from each subject, and the average CV for the five subjects was 6.1 % for the a-wave amplitude, 7.7 % for the b-wave amplitude, and 7.7 % for the sum of the oscillatory potential (OP) amplitude. The ICC was 0.76 for the a-wave amplitude, 0.68 for the b-wave amplitude, and 0.72 for the sum of the OPs amplitude. These findings indicate that our new ERG recording methods shows noise reduction and good reproducibility with low inter-session variability even with skin electrodes.

## Introduction

The electroretinogram (ERG) is used to assess the retinal function objectively in both clinical and laboratory settings [1]. ERGs can be recorded by different types of electrodes such as contact lens, conductive fibers, gold foil, conjunctival loop, corneal wick electrodes, and skin electrodes [2–6]. Among these, the electrodes which contact the corneal or bulbar conjunctiva has been commonly used, but they can cause corneal abrasions which can be painful with a potential of causing infections of the cornea. In addition, children tend not to cooperate when corneal and conjunctival electrodes are used [6–9].

In contrast, skin electrodes are less aggravating and safe, and thus obviate the need for corneal anesthesia. Skin electrodes also reduce the risk of infection and decrease the chance of mechanical trauma on corneal surface. Thus, non-invasive skin electrodes can be used in recording ERGs from children, and there has been growing interest in extending their use to other patient groups [9–17].

The amplitudes and shapes of the ERGs recorded with skin electrodes tend to be highly variable, and the amplitudes are lower than that recorded with conventional corneal electrodes [6–15]. In addition, the noise level is especially high due to the voltage changes from the lid muscles.

We have recently developed a new ERG recording system using skin electrodes (LE-4000, Tomey, Nagoya, Japan) in which the noise can be reduced. In this system, the stimulus alternates between two eyes, and each eye is stimulated several times. The voltage changes are recorded simultaneously from both eyes during the stimulation of one of the eyes, and the voltage changes of the non-stimulated eye are subtracted from that of the stimulated eye to increase the signal-to-noise ratio.

The purpose of this study was to evaluate the feasibility and reproducibility of this new ERG system with skin electrodes. First, we compared the noise levels of our ERG recording system with and without the subtraction steps in 12 normal subjects. Then, we recorded ERGs from five normal subjects on five different days to evaluate inter-session variability and reproducibility of our ERG system.

## Subjects and methods

### Subjects

A total of 17 healthy volunteers aged from 21 to 56 years were enrolled in this study, and the mean age of all subjects were 38.0 ± 12.6 (mean ± SD) years. All subjects had a best-corrected visual acuity of 20/20 or better and had no ocular diseases. The mean refractive error after spherical equivalent was −3.8 ± 2.3 diopters.

The protocol of this study was approved by the Ethics Committee of Kawasaki University of Medical Welfare (Approval number, 183). The procedures used conformed to the tenets of the Declaration of Helsinki, and they were fully explained to the subjects before the experiment, and written informed consents to participate in the study were obtained from all subjects.

### Stimulus

**Fig. 1:**
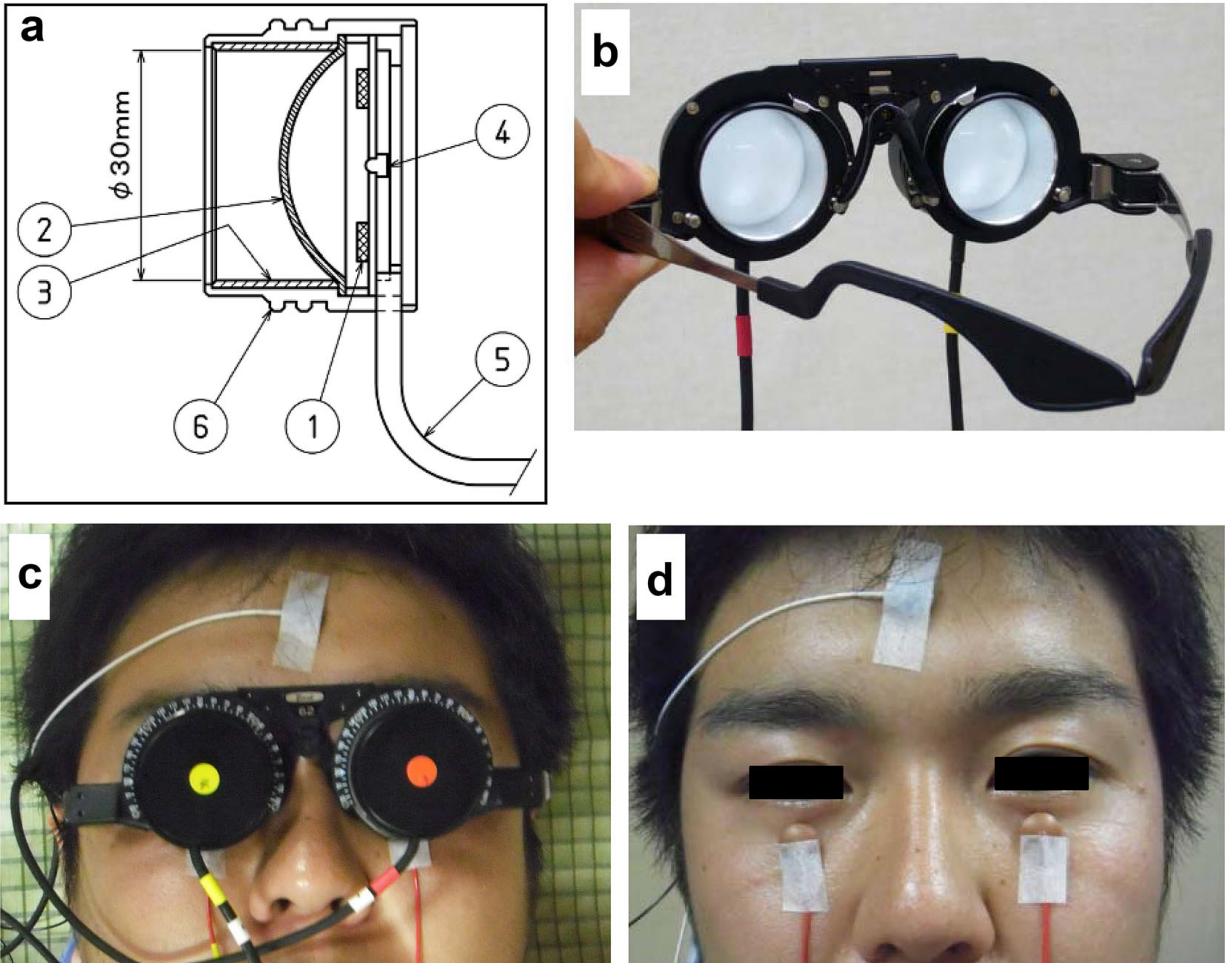
Stimulus of our ERG recording system with skin electrode. **a** Diagram of the photostimulator. *1* White LED; *2* a diffusion bulb; *3* a reflection pipe; *4* red LED for fixation lamps; *5* a power supply line of the LED. **b** The photostimulator attached to a glasses frame. **c** The subject during the recordings of the ERGs. **d** The positions of electrodes

In this study, we used only the “dark-adapted 50.0 ERG”, because we think that this dark-adapted ERG with strong flash is most informative. ERGs were recorded from both eyes simultaneously with skin electrodes using our newly developed ERG recording system. A high-intensity white LED (Nichia Corporation, Tokushima, Japan) was incorporated into a cylindrical case and served as the stimulus source (Fig. 1a). The cylindrical case contained a white hemispherical diffuser beneath the LEDs, which produced homogeneous illumination over the whole field, i.e., a Ganzfeld stimulus. Two stimulus cylinders were attached to a spectacle frame (Fig. 1b) and attached close enough to the eyes so that only one eye was stimulated (Fig. 1c). The stimulus intensity measured at the cornea was 50 cd s/m^2^ (100,000 cd/m^2^ × 0.5 ms). The stimulator also had a built-in red LED for a fixation light (Fig. 1a).

### ERG recordings

The pupils were dilated with topical 0.5 % tropicamide and 0.5 % phenylephrine, and the subjects were dark-adapted for 20 min before the recordings. The active skin electrodes were placed bilaterally on the orbital rim 7 mm from the margin of the lower eyelid (Fig. 1d). The reference electrode was placed on the midline of the forehead. These skin electrodes were commercially available, silver-plate type (Nihon Kohden, Tokyo, Japan). Electrode paste was rubbed into the skin to reduce for the impedance to less than 5 kΩ.

**Fig. 2:**
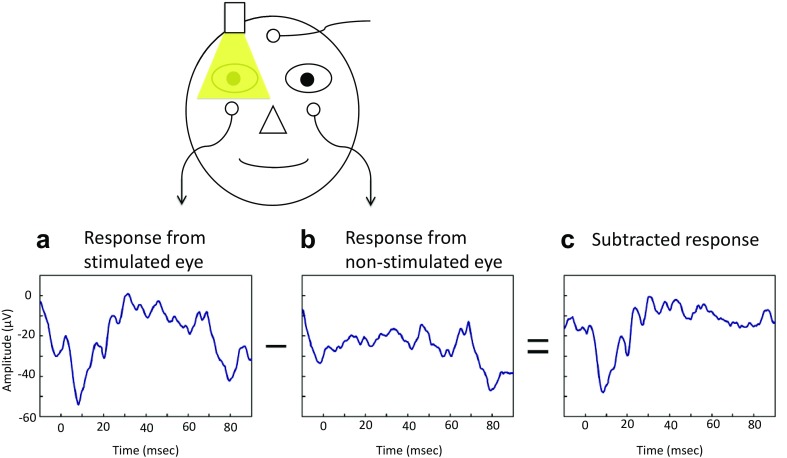
Method to increase the signal-to-noise ratio using the responses recorded from non-stimulated eye. The stimulus alternated between the two eyes every 15 s, and each eye was stimulated 8 times. The voltage changes are recorded simultaneously from both eyes during the stimulation. Then the voltage changes of the non-stimulated eye (**b**) were subtracted from that of the stimulated eye (**a**) to increase signal-to-noise ratio for each stimulus flash. Note that baseline noise was satisfactorily removed using our subtraction method in this subject (**c**)

The stimulus alternated between eyes every 15 s, and each eye was stimulated eight times. Therefore, the interval between stimuli for each eye was 30 s. The voltage changes were recorded simultaneously from both eyes during the stimulation with a half-amplitude bandwidth of 0.3–340 Hz and digitized at a sampling rate of 4 kHz. Then the voltage changes of the non-stimulated eye were subtracted from that of the stimulated eye to increase the signal-to-noise ratio for each stimulus flash (Fig. 2). Then the eight responses of each eye were averaged to further increase the signal-to-noise ratio. The overall measurement time was about 4 min. A buzzer sounded simultaneously with a flickering of the fixation light 1 s before each stimulus to alert the subject. This warning reduced blinking and eye movements during the recordings.

### Measurement of noise level

**Fig. 3:**
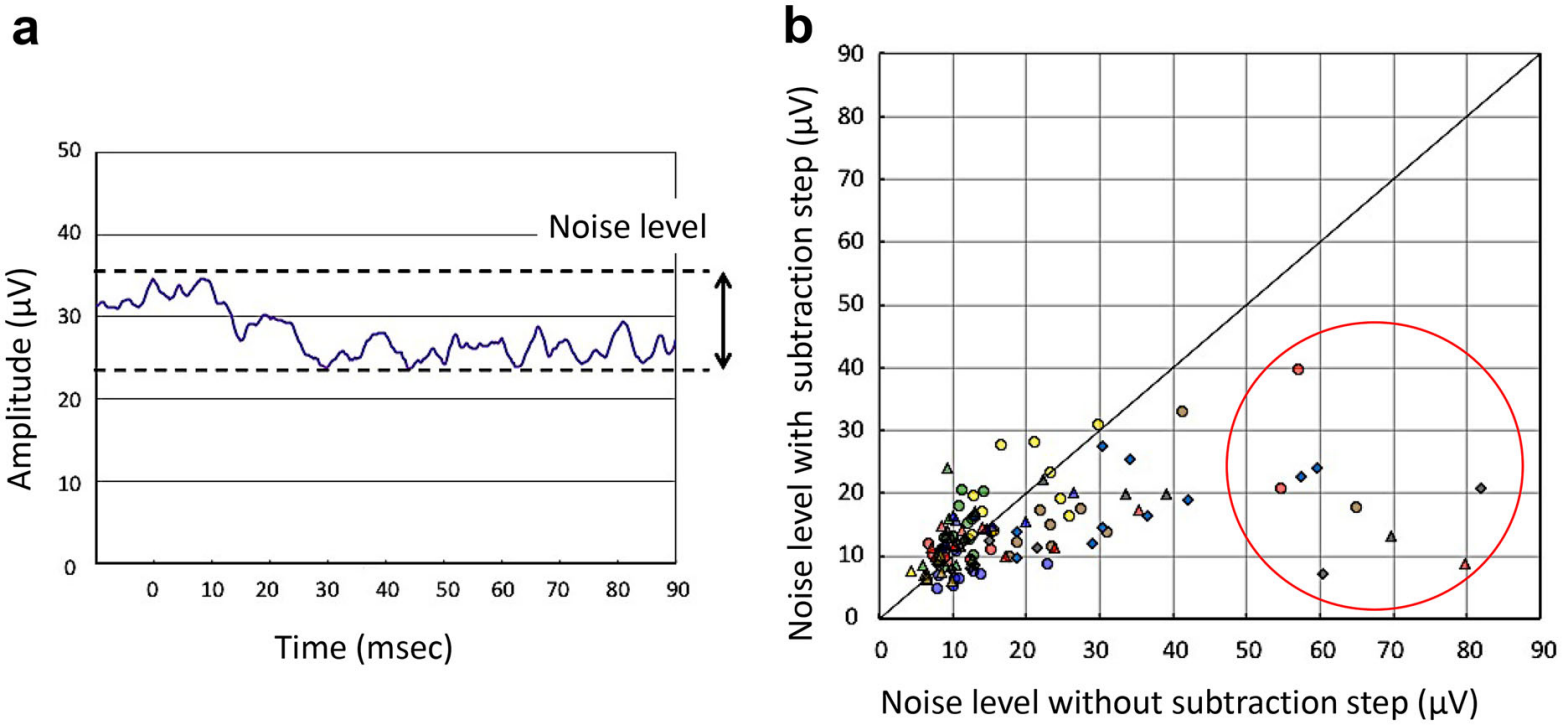
Measurement of noise level. **a** To measure the noise levels, we recorded the electrical responses without stimulus flashes, and eight responses were averaged with and without subtraction steps. Noise level was defined as peak-to-peak values during 100 ms. **b** Plot of noise levels measured five times from 12 normal subjects with and without the subtraction step. Note that the subtraction step was especially effective in reducing the noise levels when the noise levels without subtraction were higher (*red circle*). *Each symbol* shows the results of different 12 normal subjects

To measure the noise levels of our ERG recording system, we recorded the electrical responses without any stimulus flashes, and eight responses were averaged with and without subtraction steps. The noise level was defined as the peak-to-peak values during 100 ms (Fig. 3a). These noise level values were measured five times for 12 normal subjects.

### Measurement of each ERG component

The a-wave amplitude was measured from the baseline to the first negative trough. The b-wave amplitude was measured from the bottom of the a-wave to the positive peak of the b-wave. To measure the amplitude of oscillatory potentials (OPs), we measured the amplitude of each OP (O1–O4) from the peak and trough immediately preceding it. The summed OP amplitude of O1–O4 was used to assess total OP amplitudes.

### Statistical analyses

All statistical analyses were performed with SPSS^®^ Statistical software (IBM Corp., Armonk, NY). Comparison of the noise levels with and without the subtraction step was performed using paired *t* test. The coefficient of variation (CV) was expressed as a percentage and was calculated as the standard deviation divided by the mean. The intra-class correlation coefficient (ICC) was calculated with a one-way random effects model using an absolute agreement definition. The ICCs were classified as follows: ‘excellent’ (≥.81), ‘good’ (.61–.80), ‘moderate’ (.41–.60), and ‘poor’ (≤.40) according to past biometrical studies [16, 17].

## Results

### Noise level

The noise levels were measured five times from 12 normal subjects with and without the subtraction steps. All 120 values, i.e., 12 subjects × 5 times × both eyes, of noise level are plotted in Fig. 3b. In this plot, the *x*-axis represents the noise level without the subtraction step (only simple averaging), and *y*-axis represents the noise level with the subtraction step (subtraction plus averaging). We noted that the subtraction step was effective in reducing the noise levels especially when the noise levels without subtraction were higher (red circle of Fig. 3b). The mean noise level without the subtraction step was 18.4 ± 8.4 μV, and it was significantly reduced to 13.8 ± 4.0 μV with the subtraction step (*P* < 0.05).

### Amplitudes and implicit times of ERGs

**Fig. 4:**
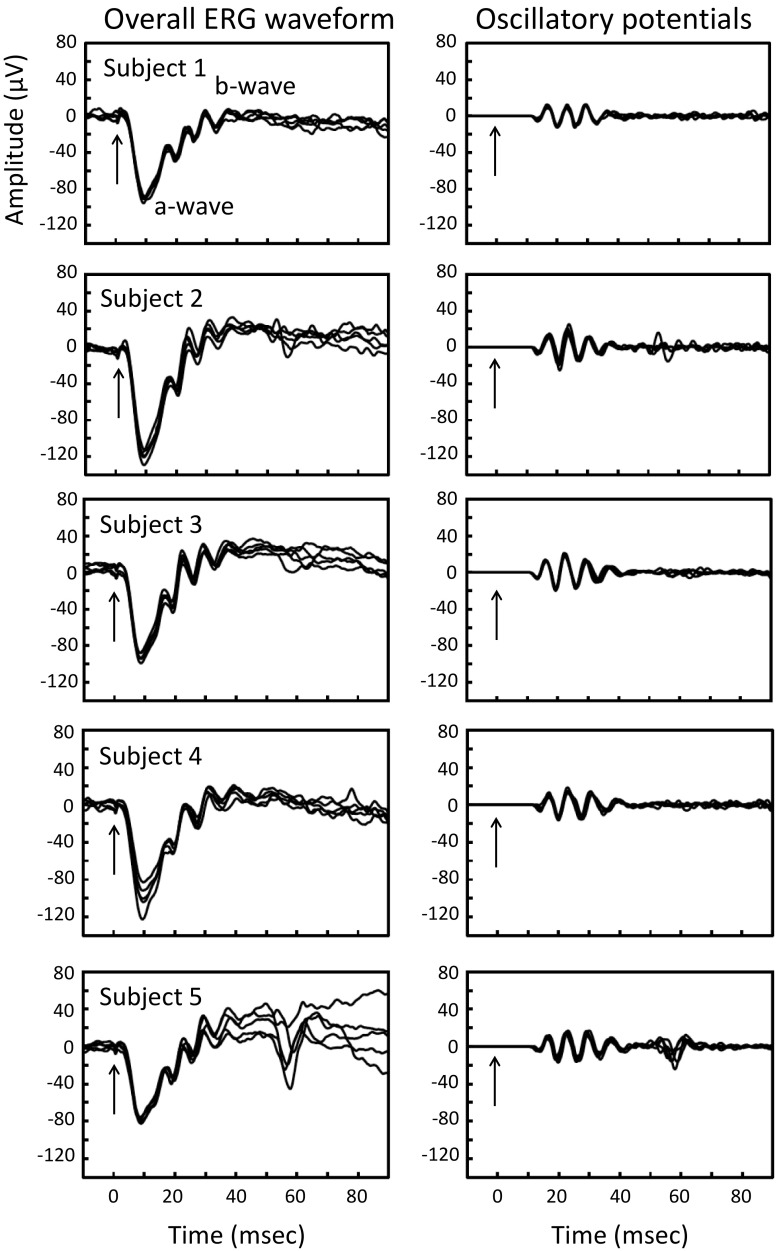
Mixed rod-cone ERGs recorded with our system using skin electrode for five normal subjects. The ERG waveforms recorded on five different days are superimposed. The ERGs are shown *on the* *left column*, and the extracted oscillatory potentials are shown *on the* *right column*

The mixed rod-cone ERGs recorded from the right eye of five subjects using our new ERG recording system with skin electrodes are shown in Fig. 4. The five ERGs recorded on 5 days are superimposed. These waveforms indicate that the inter-session variability was relatively small, and reproducibility was good.

The means (±SDs) of the amplitudes and implicit times for all ERG components, which were calculated from all 25 ERG recordings, i.e., 5 subjects × 5 days, are shown in Table 1. The means (±SDs) of the amplitudes was 96.2 ± 14.0 μV for the a-wave, 120.0 ± 20.2 μV for the b-wave, and 81.4 ± 12.4 μV for the sum of the OPs. The means (±SDs) of the implicit times was 9.2 ± 0.5 ms for the a-wave and 46.8 ± 11.2 ms for the b-wave.

**Table 1:** Summary data for each ERG component

	Amplitude			Implicit time	
	a-wave (μV)	b-wave (μV)	OPs (μV)	a-wave (ms)	b-wave (ms)
Mean	96.2	120.0	81.4	9.2	46.8
SD	14.0	20.2	12.4	0.5	11.2
Maximum	128.9	165.9	108.0	10.3	66.8
Minimum	71.3	85.3	57.7	8.0	29.3

### Inter-session variability and reproducibility

To determine the inter-session variability, the CV was calculated from the data obtained on five different days for each subject (Table 2, upper panel). The average CV for five subjects was 6.1 % for the a-wave amplitude, 7.7 % for the b-wave amplitude, and 7.7 % for the sum OP amplitude. The CV for the a-wave implicit times was 3.7 and 15.5 % for the b-wave implicit time.

**Table 2:** CV and ICC for each ERG component

	Amplitude			Implicit time	
	a-wave	b-wave	OPs	a-wave	b-wave
CV (%)	6.1	7.7	7.7	3.7	15.5
ICC	0.76	0.68	0.72	0.68	0.77
(95 % CI)	(0.43–0.97)	(0.38–0.96)	(0.43–0.97)	(0.43–0.97)	(0.43–0.97)
*P* value	0.001	0.001	0.001	0.001	0.001

*CV* coefficient of variation, *ICC* intra-lass correlation coefficients (ICCs)

Finally, we calculated the ICCs to assess the reproducibility (Table 2, lower panel). The ICC was 0.76 for the a-wave amplitude, 0.68 for the b-wave amplitude, and 0.72 for the summed OP amplitudes. The ICC was 0.68 for the a-wave implicit time and 0.77 for the b-wave implicit time.

## Discussion

Earlier studies have shown that skin electrodes can be used to record the ERGs in patients, but one major weak point was the low signal-to-noise ratio [6–18]. The ERG responses can be easily contaminated by biological noise, e.g., muscle potentials from both eyelids and the forehead. They were also contaminated by stray electrical potentials in the recording room that were picked up by both electrodes. However, these potentials are coherent or in phase so that subtracting the potential from one electrode from the other can cancel these in phase signals. Therefore, in our new ERG system, we employed this noise reduction technique by subtracting these stray potentials picked up by one eye from that of the other eye which had picked up the same voltage changes. The results showed that the noise level was significantly reduced when the subtraction step was added. This noise reduction method was more effective when the basic noise levels were higher (Fig. 3b). Thus, it was possible to detect the OPs picked up from a skin electrode affixed to the lower lid with our method (Fig. 4). We have also recently reported that both the amplitude and implicit times of our skin–electrode ERG system were significantly correlated with those recorded with conventional ERG systems with corneal electrodes [18].

We found that the inter-session variability was considerably small for both amplitude and implicit times in our system. The average CV values of the ERG amplitudes were less than 10 % for all components. A search of PubMed using key words of “coefficient of variations of normal ERGs” and “inter-session variability of ERGs” did not extract any publications. Thus, we were not able to compare our data to any past data. The average CV of the ERG implicit times was small for the a-wave (3.7 %), but was larger for the b-wave (15.5 %) (Table 2). This larger value can be explained by the fact that the b-wave peak is broad when compared to the a-wave or OPs.

We also calculated the ICCs for the assessment of reproducibility. The ICC was between 0.68 and 0.77 for both amplitudes and implicit times of all ERG components (Table 2), suggesting that the reproducibility of our system is ‘good’ according to past biometrical criteria [16, 17]. However, we cannot conclude whether the reproducibility of our ERG system is really good, because we did not compare the ICCs of our system with other ERG recording systems. In addition, we could not find any past papers on the ICC values for other ERG systems. Further studies are needed to compare the reproducibility of our system with other ERG recording systems.

Because the position of the electrode is relatively far from the eye compared to the standard corneal electrode, the amplitudes of the ERG recorded with the skin electrode are smaller (Table 1) which is in agreement with earlier studies [6–10, 13]. The amplitudes of the ERG responses recorded with skin electrodes were about one-quarter to one-fifth of the responses recorded with the corneal-contact electrodes. The differences in the degree of reduction may be related to differences in electrode position/application as it has been reported that electrode positioning has a marked effect on the amplitude of responses recorded by skin electrodes [8].

One of the inherent drawbacks with the skin electrode ERG is that the data acquisition time is longer than the conventional ERG, because the response amplitude is small and requires averaging of the data. We may be able to shorten the acquisition time by decreasing the number of measurements in subjects without significant noise, depending on the quality of the data. Another problem with this technique is that ERGs can be elicited with the subject’s eyes closed, and thus data may be recorded during a blink. However, the waveform acquired during the blink can be removed manually. Third drawback with this ERG system is that we cannot check the subjects’ fixation during the recording.

In conclusion, our findings suggest that ERGs recorded with skin electrodes can be highly reproducible with low -noise level and can be used to assess the integrity of the retinal function. Our ERG recording system can be used for children, reduce the risk of infection, and be safe for the post-operative patient after intraocular surgery.
